# Colonoscopy Trainers Experience Greater Stress During Insertion than Withdrawal: Implications for Endoscopic Curricula

**DOI:** 10.1093/jcag/gwz031

**Published:** 2019-11-23

**Authors:** Madeline Lemke, Alison Banwell, Natalie Rubinger, Michelle Wiepjes, Mark Ropeleski, Stephen Vanner, Lawrence Hookey

**Affiliations:** Gastrointestinal Diseases Research Unit, Queen’s University, Kingston, Ontario, Canada

**Keywords:** Colonoscopy, Endoscopy, Medical education, Simulation, Training

## Abstract

**Background:**

Optimal colonoscopy training curricula should minimize stress and cognitive load. This study aimed to determine whether withdrawal or insertion colonoscopy skills training is associated with less stress or cognitive load for trainees or trainers.

**Methods:**

In Phase I, participants were randomized to train on either insertion or withdrawal in a simulated environment. In Phase II, participants were randomized to begin with either insertion or withdrawal in patient encounters. Salivary cortisol levels, heart rate, and State-Trait Anxiety Inventory (STAI) surveys were used to assess stress in trainees and trainers. NASA Task Load Index (TLX) survey was used to assess cognitive workload in trainees.

**Results:**

In Phase I, trainee stress increased during the simulation training during both withdrawal and insertion compared to baseline, while trainer stress changed minimally. Cognitive load was higher for trainees during withdrawal (*P* = 0.005). In Phase II, trainers’ STAI scores were greater during insertion training (*P* = 0.013). Trainees’ stress was highest prior to beginning patient training and decreased during training, while trainer’s stress increased during training. Trainees reported insertion training being of greater value (70.0%), while trainers reported withdrawal was preferred (77.8%).

**Conclusion:**

Trainees and trainers exhibit important differences in stress during colonoscopy skills training. Trainees reported more stress during simulation training and greatest cognitive load during simulation withdrawal, whereas trainers reported greatest stress during patient encounters, particularly training of insertion techniques. Attention to the effect of stress on trainees and trainers and the drivers of stress is warranted and could be incorporated in competency based medical education.

## Introduction

Colonoscopy is a technically challenging procedure that requires skilled endoscopists to deliver safe and effective care (1). There are many components of colonoscopy training, some of which can invoke stress and increase cognitive load, potentially limiting performance and learning (2). Modern training in colonoscopy involves both simulation and patient-based training, and could be further enhanced by thoughtfully structured curricula in order to reduce both undue trainee and trainer stress and optimize skills acquisition (1,3,4). An important part of gastroenterology training programs is tailoring colonoscopy teaching to the appropriate level of the learner in order to optimize success in acquiring colonoscopy skills (3,4).

Technical skills that have been identified as important components of colonoscopy training curricula include torque steering, loop reduction and appropriate visualization of the mucosa and lumen (3,5). One variable that has not been studied is whether there is an advantage to begin learning during withdrawal of the colonoscope compared to insertion. During withdrawal the focus is on scope tip control, maintaining the luminal view, and lesion detection. During insertion, a process that carries an increased risk of perforation, additional skills in navigation are demanded of the learner. Theoretically, the latter could overwhelm the novice learner or their trainer, while others may find the process associated with withdrawal challenging. This study sought to determine whether colonoscopy training that initially focuses on colonoscope withdrawal compared to insertion leads to lower stress levels, lower cognitive workload, and increased perceived helpfulness in skills acquisition for both trainees and trainers.

## Materials and Methods

Trainees were internal medicine residents with no prior endoscopy experience. The trainers were experienced gastroenterologists, each of whom had completed over 1000 endoscopic procedures, had completed a formal ‘*train the trainer*’ colonoscopy course, and had been training gastroenterology fellows for over 10 years (6). All trainees were given a standard simulation laboratory teaching session on the basics of colonoscopy and scope handling prior to the active study component by a CAG Skills Enhancement in Endoscopy certified trainer (L.H.). The Queen’s University Health Sciences Research Ethics Board approved the study. It was performed in two phases, with Phase I consisting of colonoscopy simulation training and Phase II consisting of colonoscopy training during patient encounters. Different participants were recruited in Phase I and Phase II.

### Assessment Tools

Stress level and cognitive workload were examined in trainees and stress level was examined in trainers. Stress was assessed using the Imperial Stress Assessment Tool (ISAT), which includes three components: salivary cortisol levels, heart rate, and the self-reported State-Trial Anxiety Inventory (STAI) (7,8). The ISAT provides a feasible, nonintrusive method to assess stress levels without impacting routine practice (7). Cortisol was extracted from saliva captured in a cotton swab prior to and immediately following a training exercise, and was reported in nmol/L. Differences in heart rate were reported as the change from baseline heart rate and maximum heart rate during the procedure in beats per minute (bpm). The devices used to measure heart rate were worn on trainees’ and trainers’ forearms with data continuously uploaded and recorded on a wireless device (Rhythm armband heart rate monitor, Scosche Industries Inc., Oxnard, CA). STAI scores were calculated at baseline and after the insertion/withdrawal procedure was completed. All measures of stress were reported as the difference between baseline and the period immediately following the procedure, with a positive number indicating an increase in stress over the course of the procedure, a negative number indicating decreased stress and zero indicating no change in stress.

Cognitive load was measured using the NASA Task Load Index (NASA-TLX), a rating scale that assesses the physical demand, effort, performance and educational capacity in performing tasks (9,10). A higher number indicates an increased cognitive load required to complete the task. Trainees and trainers were also asked to report whether insertion or withdrawal was perceived to be a more valuable training session.

### Phase I—Clinical Simulation

In Phase I, trainees were randomized to train on either insertion or withdrawal of adult colonoscopes (Pentax EC 3890Li, Pentax Medical, Mississauga, Ontario, Canada) using clinical silicone colonoscopy simulators (M40 colon simulator, Kyoto Kagaku America Incorporated, CA). Trainees were given a maximum of 20 minutes to perform either insertion or withdrawal with stress and cognitive workload assessed as described above.

### Phase II—Patient Encounter

In Phase II, trainees were trained to perform colonoscopy on patients after standardized simulation lab teaching. All trainees were trained on insertion and withdrawal; however, they were randomized to determine which component they trained with first. Both sessions were completed on the same day. Trainee demographics were collected including their training level and previous exposure to endoscopy or endoscopy simulation. Trainees were given 20 minutes to perform insertion or withdrawal tasks contingent on the patient remaining comfortable and safe in the opinion of the trainer. Trainees’ stress, cognitive workload, and whether they found insertion or withdrawal more valuable to train on was assessed. Trainers’ stress and whether they found insertion or withdrawal more valuable training was also assessed. In Phase II, stress and cognitive load after withdrawal versus insertion training was compared.

### Analysis/Statistics

Statistical significance was defined as α = 0.05. In Phase I, independent *t*-tests were used to compare between results of withdrawal and insertion training for both trainees and trainers. In Phase II, paired *t*-tests were used to compare between withdrawal and insertion training for both trainees and trainers. All analyses were performed using SAS Version 9.4 (SAS Institute Inc., Cary, NC).

The difference in State-Trait Anxiety Inventory was used to determine the sample size required based on the previous findings of Arora et al. which showed a statistically significant difference (*P* < 0.05) in postoperative STAI of 9.81 (SD 2.20) in nonstressful cases to 12.87 (SD 4.27) in stressful cases (7). Using an α of 0.05 and a power of 0.80, a minimum of 16 trainees were required for Phase I to detect difference in STAI of 3. The sample size for Phase II was calculated using paired analysis, and a minimum sample size of 8 subjects was needed to detect a difference of 3 in the STAI, with an assumed SD of 3 (7).

## Results

### Phase I—Clinical Simulation

**Figure 1.**
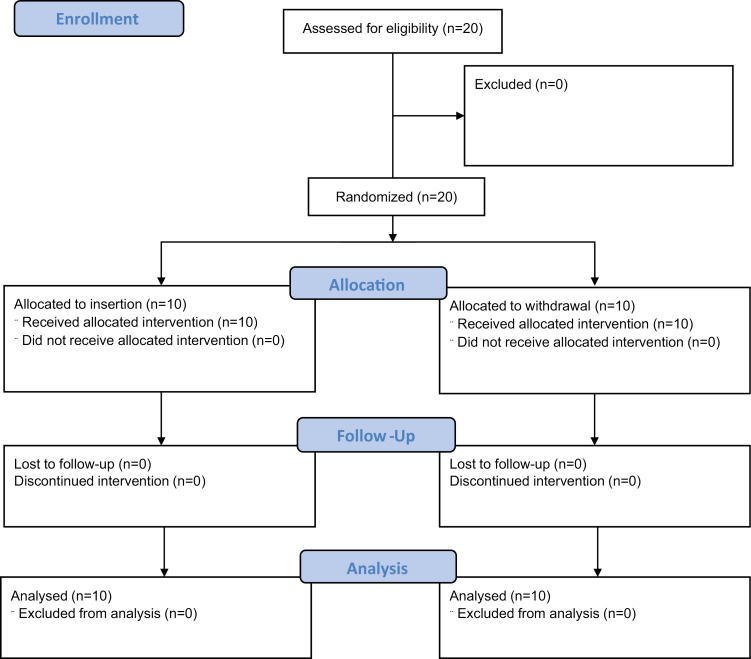
Phase I participant flow map.

A total of 20 trainees and 6 trainers were included in Phase I (Figure 1), with ten trainees randomized to withdrawal training and ten randomized to insertion training.

Among trainees, all measures of stress increased from baseline following the procedure as indicated by positive delta values (cortisol, maximum heart rate, STAI score); however, no differences between withdrawal and insertion were detected (*P* = 0.774, *P* = 0.503, *P* = 0.289; Table 1). Cognitive load was greater during withdrawal compared to during insertion (310.80 versus 235.00, *P* = 0.005).

**Table 1.** Stress and cognitive load in simulated colonoscopy training (Phase I)

	Withdrawal (*n* = 10)	Insertion (*n* = 10)	*P*-value
Trainees			
**Difference between cortisol before and after procedure (nmol/L)** Mean (SD)	2.63 (1.30)	2.25 (0.98)	0.774
**Difference in maximum heart rate (bpm)** Mean (SD)	9.78 (13.20)	5.25 (10.80)	0.503
**Difference between STAI score before and after procedure** Mean (SD)	3.61 (−0.80)	2.41 (0.70)	0.289
**NASA-TLX score after procedure** Mean (SD)	310.80 (37.89)	235.00 (61.85)	0.005*
**Trainers**			
**Difference between cortisol before and after procedure (nmol/L)** Mean (SD)	−0.57 (1.19)	0.52 (1.05)	0.126
**Difference in maximum heart rate (bpm)** Mean (SD)	14.67 (15.91)	7.00 (6.45)	0.300
**Difference between STAI score before and after procedure** Mean (SD)	0.00 (1.10)	0.33 (1.63)	0.687

bpm, beats per minute; NASA-TLX, NASA Task Load Index; SD, standard deviation; STAI, State Trait Anxiety Inventory.

*Indicates statistical significance (P ≤ 0.05).

Among trainers, cortisol and STAI score showed only minimal change from baseline for both withdrawal and insertion arms. Maximum heart rate increased during the procedure in both arms but no significant differences were detected between withdrawal and insertion groups.

### Phase II—Patient Encounter

**Figure 2.**
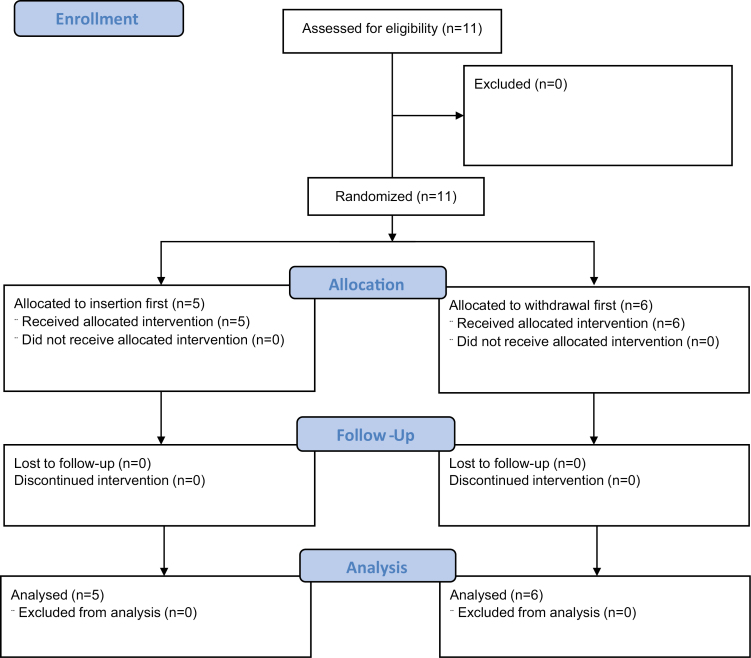
Phase II participant flow map.

A total of 11 trainees were included in Phase II (Figure 2), with 6 randomized to begin with withdrawal and 5 to begin with insertion. Three trainers (who also participated in Phase I) participated in Phase II.

Among trainees, cortisol levels were lower following the procedure compared to before, with no differences between insertion and withdrawal arms (*P* = 0.152; Table 2). Maximum heart rate showed minimal change in the withdrawal arm but increased in the insertion arm with no statistically significant difference detected between the two groups (*P* = 0.259). STAI scores changed minimally in both arms, with no difference detected between the two arms (*P* = 0.517). No differences in cognitive load between the two arms were detected (*P* = 0.065). The majority of trainees reported a greater value with insertion training (70.0%).

**Table 2.** Stress and cognitive load during clinical patient colonoscopy training (Phase II)

	Withdrawal (*n* = 11)	Insertion (*n* = 11)	*P*-value
**Trainees**			
**Difference between cortisol before and after procedure (nmol/L)** Mean (SD)	−3.28 (3.46)	−1.71 (2.21)	0.152
**Difference in maximum heart rate (bpm)** Mean (SD)	0.14 (9.15)	11.00 (22.35)	0.259
**Difference between STAI score before and after procedure** Mean (SD)	−0.18 (3.63)	0.45 (3.42)	0.517
**NASA-TLX score after procedure** Mean (SD)	239.73 (54.18)	272.45 (55.72)	0.065
**Trainers**			
**Difference between cortisol before and after procedure (nmol/L)** Mean (SD)	2.79 (1.63)	3.02 (2.45)	0.784
**Difference in maximum heart rate (bpm)** Mean (SD)	8.70 (17.10)	−1.27 (16.61)	0.064
**Difference between STAI score before and after procedure** Mean (SD)	1.70 (2.06)	3.64 (3.56)	0.013*

bpm, beats per minute; NASA-TLX, NASA Task Load Index; SD, standard deviation; STAI, State Trait Anxiety Inventory.

*Indicates statistical significance (P ≤ 0.05).

Among trainers, cortisol levels increased following the procedures with no difference between the withdrawal and insertion arms (*P* = 0.784). STAI scores also increased following the procedures, but with a greater increase in the insertion arm (*P* = 0.013). Maximum heart rate increased in the withdrawal arm but decreased slightly in the insertion arm, with no statistically significant difference detected. The majority of trainers (77.8%) reported greater value with withdrawal training.

## Discussion

The current study evaluated whether colonoscopy skills training during insertion versus withdrawal affects the learning and teaching experience for trainees and trainers. Stress and cognitive load were the foci of the analysis. The only statistically significant differences detected between withdrawal and insertion tasks were the increased cognitive load for trainees during withdrawal in simulation and increased stress (as measured by STAI scores) for trainers during patient insertion. Notably, stress patterns differed between trainees and trainers as well as between simulation and clinical patient encounters. During simulation, trainees’ stress increased through the procedure while trainers’ stress remained unchanged. During clinical patient encounters, trainees’ stress was greatest prior to beginning the training session and decreased throughout the procedure, while trainers’ stress increased through the patient training sessions.

An aspect of medical education that has not yet been extensively studied is the stress experienced by the trainee in various learning scenarios, and the effect this may have on their skill acquisition (11). Even less research has been done with respect to trainer stress during direct teaching. The development of *train the trainer* type courses has been met with appreciation and widespread uptake, yet quantification of the stress of training in various endoscopy situations has yet to be described (6). In this study, when trainees were trained to perform colonoscopy during simulation, their stress increased through the procedure. This may suggest that trainees felt comfortable in the simulated environment, but their stress increased once facing the challenges and demands of the task. Trainers, however, did not show increasing stress throughout the simulated procedure. Conversely, during clinical encounters with patients, trainee stress decreased during the procedure while trainer stress increased. This suggests that trainees may feel more stress prior to initiating colonoscopy on patients that is alleviated on completion; whereas trainers feel increased stress when teaching colonoscopy during patient encounters. These patterns indicate unique challenges and obstacles to endoscopy training for both trainees and trainers in simulated and clinical environments that have not been previously described. Further studies are needed to examine what drivers contribute to these differing stress patterns.

In the last decade, much of the focus in teaching of endoscopy skills has shifted from volume thresholds to competency assessment (12–16). The progress of trainees has been tracked and, not surprisingly, the rate of achievement of competency is variable. Further evolution of endoscopy education research in this field has focused on how to teach these skills, particularly integration of simulators and models (3,4). Results definitively demonstrate that active teaching during simulation results in improved skill acquisition compared to trainees left alone on the simulator thus translating directly to improved clinical colonoscopy skills (3,4). However, little data exist on the trends of stress that exist for trainees and trainers during simulation training and if this can be used as an indicator of whether trainees can graduate onto clinical encounters with patients.

There are some limitations to this study. Cognitive load was assessed using the NASA-TLX; however, a colonoscopy specific tool exists (17). This study did not to utilize the CLIC tool, as it contains patient case specific factors which would not translate well to simulated colonoscopy. Because of this, NASA-TLX was utilized for both simulated and patient colonoscopy for uniform results. The CLIC tool can identify sources of increased cognitive load, and this added value was lost in the current study. Multiple outcomes were examined, and the study was unfortunately not powered to ensure all outcomes could be sufficiently examined with the sample size of trainees and trainees studied. Thus, it remains unclear whether the lack of statistically detected difference represents a true negative result or an underpowered objective. In addition, performance was not measured and increased stress for either trainees or trainers cannot be correlated with trainee performance. Similarly, metrics on the complexity of patient cases was not examined making it unclear whether increased stress or cognitive load could be attributed to more challenging cases.

This study is strengthened by use of both objective (salivary cortisol, heart rate) and subjective (STAI) measures of stress to give a more robust stress assessment. A difference in stress for initiating colonoscopy training with withdrawal versus insertion was not identified, however, different patterns of stress were observed for trainees and trainers in simulated and clinical encounters. The study did have a short timeline, where participants only performed one or two procedures and were not examined at later timepoints. As learning a new technical skill requires time and repetition, it is tempting to speculate that a longer study may find significant changes in stress outcomes as participants become more adept at colonoscope insertion and withdrawal. It also would be interesting to note if changing trainers between timepoints would affect both the trainee and trainer experience. Examining the participants over a course of repeated learning events could provide information concerning the learning curve and rates of skills acquisition, and how these factors relate to the objective and subjective measures of stress over time. The Challenge Point Framework highlights that learning cannot occur without information, but that excessive information impedes learning (18). Stress and cognitive load may have a role in obtaining this balance, by perhaps assisting in identifying competency or suggesting graduating to more complicated skills or more clinical exposure (18).

Trainees perceived training on colonoscopy insertion to be of greater value (70.0%) while trainers perceived withdrawal training to be of greater value (77.8%). The explanation for these major differences is not clear. It may highlight the importance of communication of training goals between the trainee and trainer prior to starting the procedure. Increased communication of goals may focus the trainee on the tasks expected by the trainers and enhance their learning and performance.

This study investigated whether differences in variables such as trainee stress, trainee cognitive load and trainer stress could enlighten the choice to teach withdrawal skills or insertion skills during novice colonoscopy skills acquisition. A superior method was not identified; however, unique patterns of stress were observed for trainees and trainers. Trainees experienced increased cognitive load during the withdrawal phase of simulation training, whereas trainers experienced greater stress during colonoscope insertion training during patient encounters. Trainees found the simulated environment initially less stressful than the intraprocedure phase; however, they found clinical patient encounters initially stressful with reduced stress through the procedure. Simulated training did not affect trainer stress, however, patient encounters increased trainer stress. Future research directed at identifying drivers of stress and strategies to mitigate stress for trainees and trainers as well as the relationship between stress and performance is warranted. This could enhance learning acquisition and could perhaps be incorporated as a component of competency based medical education.

## Funding

This research was supported by a Southeastern Ontario Medical Association Clinical Scientist Award and the Educational Innovation and Research Fund

## Conflicts of Interest

We have no conflicts of interest to disclose. This research was supported by a Southeastern Ontario Medical Association Clinical Scientist Award and the Educational Innovation and Research Fund.

## Notes

Part of this work has been presented at the Canadian Digestive Disease Week in Toronto, Ontario in (February 2018) and Digestive Disease Week in Washington, D.C. (June 2018)
